# Heavy Silicone Oil as a Long-Term Endotamponade Agent for Complicated Retinal Detachments

**DOI:** 10.1155/2014/136031

**Published:** 2014-04-15

**Authors:** Juliana Prazeres, Octaviano Magalhães, Luiz F. A. Lucatto, Rodrigo Milan Navarro, Nilva S. Moraes, Michel E. Farah, André Maia, Maurício Maia

**Affiliations:** ^1^Vitreoretinal Diseases Unit, Department of Ophthalmology, Federal University of São Paulo, Botucatu Street 816, 04023-062 São Paulo, SP, Brazil; ^2^Brazilian Institute of Fight Against Blindness, Rua Botucatu 816, Otto Ribeiro Avenue 901, 19814-050 Assis, SP, Brazil

## Abstract

We retrospectively evaluated a heavy silicone oil (HSO) as a long-term intraocular endotamponade agent to treat complicated RD by inferior PVR in 25 eyes of 25 patients. Patients underwent PPV and injection of Oxane HD as an internal tamponade agent. A comparison of preoperative and postoperative BCVA at month 1, month 6, and last visit was made in the group in which HSO was removed and in the group in which HSO was not removed. Statistical calculations were performed using the Wilcoxon test. The HSO was removed from 11 patients after a mean of 26.55 ± 21.38 months. The HSO remained inside the vitreous cavity in 14 eyes due to a high chance of PVR recurrence (mean follow-up period, 11.07 ± 7.44 months). Anatomic success was achieved in 92%. The BCVA in the group, in which HSO was not removed, improved significantly during the first 6 months. Among the patients who had the oil removed, there was improvement in BCVA after 1 month. Oil emulsification was the most common adverse effect in 52% of eyes. HSO is an effective tamponade in complex rhegmatogenous and tractional RD complicated by PVR. HSO can remain in the eye for long periods with relative tolerability and safety.

## 1. Introduction

Silicone oil is an excellent endotamponade agent for superior breaks and detachments complicated by proliferative vitreoretinopathy (PVR). However, its density, which is lower than water, may result in fluid accumulation in the inferior quadrant, which is not covered by silicone oil, and ineffective tamponade at the retinal breaks [1–3]. Therefore, an aqueous environment with inflammatory and cellular proliferation may promote development of inferior PVR [4–7]. Despite improvement of vitreous microsurgical techniques, the surgical treatment of PVR is challenging in vitreoretinal surgery and can lead to blindness and ocular globe atrophy [3].

A high-density silicone oil was developed [3, 8–12] as an endotamponade agent for use in cases of complicated retinal detachments, especially those with inferior PVR.

Heavy silicone oil (HSO) has a high density and is heavier than water. Due to the properties of HSO, it has been proposed for use in treating complicated retinal detachments [3, 13, 14]. Oxane HD (Bausch & Lomb, Rochester, NY, USA) is a HSO comprised of a mixture of ultrapurified silicone oil (Oxane 5,700 centistokes) and RMN3 (partly fluorinated olefin). The mixture, with a density of 1.02 g/cm^3^, a viscosity of 3,300 mPas, and a refractive index of 1.40, is homogeneous and stable in the presence of water and air and its surface tension is higher than 40 mN/m [2, 3].  Table 1 shows the HSO chemical and physical properties.

Some authors have reported that HSO is associated with ocular inflammatory reactions, increased intraocular pressure (IOP), cataract formation, and emulsification as well as difficulties to remove the HSO from the eye [15–17].

Several published studies have analyzed the complications and anatomic success rates after short-term intraocular tamponade with HSO. In most studies, the HSO was removed between 3 and 6 months postoperatively.

The aim of the current study was to evaluate the anatomic outcomes, functional results, and ocular adverse effects in patients with complicated retinal detachments in whom Oxane HD was used as a long-term intraocular endotamponade agent.

## 2. Methods

We retrospectively studied 25 eyes of 25 patients with complicated retinal detachments by PVR. Patients underwent pars plana vitrectomy (PPV) and injection of Oxane HD as an internal tamponade agent. The surgeries were performed between 2006 and 2013 in the retina sector of the Federal University of São Paulo, São Paulo, and the Brazilian Institute of Fight Against Blindness, Assis, São Paulo, Brazil.

The inclusion criteria were primary or recurrent rhegmatogenous retinal detachments complicated by inferior PVR worse than CA3 [18] and/or complicated by hypotonia in eyes with combined rhegmatogenous and tractional retinal detachment associated with PVR that occurred in the context of diabetic retinopathy associated with retinal breaks due to severe fibrovascular proliferation. All retinal detachments in this study were considered to have a poor prognosis and the probable outcome should be the globe atrophy due to ciliary body traction related to advanced PVR. The follow-up period was at least 6 months.

The exclusion criterion was the presence of a severe systemic disease or inability to undergo regular follow-up examinations.

Patients were advised that the injection of HSO was based on published experience of 3-to-6-month use of this silicone oil as an endotamponade agent in inferior PVR. They were informed that, due to the complex clinical situations and the poor prognosis of the study eyes, HSO was used as a vitreous substitute for longer than the current reported time in the literature. All patients provided their informed consent and have authorized the use of their clinical data in the study.

The preoperative and postoperative data included the medical history, measurement of the BCVA using a Snellen chart, slit-lamp examination, intraocular pressure (IOP) measured by Goldmann tonometry, binocular fundoscopy, B-scan ultrasonography, and fundus photographs.

Follow-up examinations were scheduled for postoperative day 1, week 1, and months 1 and 3 after the initial surgery and every 3 months until the end of the follow-up period. Unscheduled appointments, complications, and additional interventions were documented.

The same vitreoretinal surgeon (Maurício Maia) performed all surgeries using local retrobulbar anesthesia. The surgery included a standard three-port, 23-gauge PPV, phacoemulsification. Retinotomy, retinectomy, and internal limiting peeling were performed if necessary. Endophotocoagulation was performed to treat retinal breaks. Scleral buckling was performed following retinotomies of 180 degrees or more or if there was residual vitreous at the vitreous base at the end of the surgical procedure.

Direct perfluorocarbon-silicone exchange was avoided to prevent “sticky oil” formation; in all patients, the perfluorocarbon liquid was aspirated completely due to a fluid air exchange followed by injection of Oxane HD (HSO) under air.

When the HSO was removed, it was aspirated using a 19-gauge needle BD (Becton Dickinson, USA) connected to an extrusion silicon tube; the needle was inserted by sclerotomy via pars plana and the extrusion silicon tube was changed 2-3 times due to obstruction of the system by the HSO. Many times, a bubble of residual silicon oil was deposited at the posterior pole and such technique of HSO removal is important information for vitreoretinal surgeons that will perform this surgical technique.

The preoperative and final postoperative BCVA levels were analyzed after they were converted to the logarithm of the minimum angle of resolution (logMAR).

The study adhered to the tenets of the Declaration of Helsinki and all federal laws. The ethics committee of our institution approved the study.

A comparison of preoperative and postoperative best-corrected visual acuities at month 1, month 6, and last visit was made in the group in which HSO was removed and in the group in which HSO was not removed. Statistical calculations were performed using the Wilcoxon test to compare the preoperative and postoperative VA levels. *P* < 0.05 was considered statistically significant. The SPSS (v15.0) statistical package was used for statistical analysis.

## 3. Results

Twenty-five eyes of 25 patients (19 men, 6 women; mean age, 49 ± 18.2 years; range, 17 to 80 years) were included in this study. The surgeries were performed between March 2006 and June 2013. The mean follow-up time was 21.44 ± 15.28 months.

Seventeen eyes had a rhegmatogenous retinal detachment complicated by inferior PVR; eight eyes had a tractional retinal detachment due to proliferative diabetic retinopathy complicated by retinal breaks and inferior PVR. All eyes included in this study had a macular detachment and also hypotony.

Among the eyes with a rhegmatogenous retinal detachment, one was secondary to toxoplasmosis uveitis, another had a complicated retinal detachment secondary to trauma, and the last one had multiple angiomas secondary to Von Hippel-Lindau disease.

The retinal detachments in all eyes were considered to have a poor prognosis due to the presence of advanced PVR.  Table 2 shows the detailed patient data and the classifications of PVR [18].

Oxane HD was the primary tamponade agent in eight (32%) eyes. Seventeen (68%) patients had undergone a previous unsuccessful surgery for retinal reattachment and underwent retreatment with Oxane HD due to development of severe PVR after the first surgery. During the first surgery, PPV with injection of octafluoropropane gas (C3F8) was performed in five of these eyes, 1,000-centistoke silicone oil was injected in 10 eyes, and 5,000-centistoke silicone oil was injected in two additional eyes. All the 12 eyes that received silicone oil during the first surgery had a redetachment despite use of an endotamponade agent.

Two eyes (eyes 11 and 16) had undergone a previous PPV associated with scleral buckling in another institution. In three other eyes (12, 13, and 23), scleral buckles were implanted during the retreatment.

Sixteen eyes were phakic and nine were pseudophakic. Among the phakic patients, 14 underwent cataract extraction associated with PPV and two underwent cataract extraction when the HSO was removed.

The HSO was removed from 11 eyes after a mean period of 26.55 ± 21.38 months. In these eyes, the IOP became elevated in four (16%) eyes during the follow-up period. The IOP was uncontrolled in three patients despite instillation of antiglaucomatous eye drops and the HSO was removed and replaced with a 5,000-centistoke silicone oil. The retina remained stable and reattached in all eyes after the HSO was removed. Additional procedures during HSO removal included phacoemulsification (2 eyes), epiretinal membrane (ERM) peeling (1 eye), scleral IOL implantation (1 eye), and secondary IOL implantation (1 eye).

The HSO was left in the eyes of 14 (56%) patients because of the high risk of recurrence of the retinal detachment and ocular globe atrophy. Among these eyes, one had an oil drop in the anterior chamber; however, we elected not to remove the HSO due to the poor prognosis. In another eye, the HSO was not removed due to superior persistent retinal detachment and PVR. Three of these patients needed topical antiglaucomatous eye drops to control the IOP. The mean follow-up period was 11.07 ± 7.44 months.

**Figure 1:**
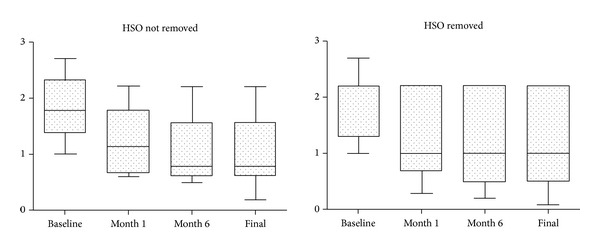
LogMAR BCVA Box plot at baseline, month 1, month 6, and final in patients in whom HSO was not removed and in patients in whom HSO was removed.

The mean preoperative logMAR BCVA in the group in which HSO was not removed was 1.90 ± 0.58, which increased significantly to 1.22 ± 0.58 at month 1 (*P* = 0.001) and to 1.07 ± 0.63 at month 6 (*P* = 0.027). Between 6 months and the last visit of follow-up, there was no statistically significant difference in the analysis of BCVA. In the group in which HSO was removed, the mean baseline logMAR BCVA was 1.87 ± 0.63 which increased significantly to 1.33 ± 0.74 at month 1 (*P* = 0.008). Between the first month and the last visit, there was no statistically significant difference in BCVA (Table 3 and Figure 1).

Ten (40%) eyes had cells in the anterior chamber without hypopyon or keratic precipitates during the first postoperative month. Inflammatory reactions resolved in all eyes with topical steroids within 15 days.

Oil emulsification occurred in 52% of the eyes. Complications such as development of an ERM during Oxane HD tamponade occurred in one (4%) eye. Lens opacity progressed in all phakic patients, and they underwent cataract surgery at the same time the HSO was removed.  Table 2 lists the other complications.

Anatomic success was achieved in 92%. One eye had a recurrence of the rhegmatogenous retinal detachment and another eye had a persistent tractional retinal detachment. No additional surgeries were performed in these cases due to the poor prognosis and risk of ocular globe atrophy.

## 4. Discussion

In this retrospective study, we described the effects of HSO as an endotamponade agent for complicated retinal detachments with inferior PVR. Despite advances in PPV techniques, vitreoretinal surgeons are still challenged by complex retinal detachments complicated by PVR.

Standard silicone oil is an excellent tamponade agent for most retinal detachments [4, 19, 20]. However, the tamponade of the inferior retina may be unsatisfactory since the density of standard silicone oil is lower than water. This results in an aqueous inflammatory environment that may predispose and increase the possibility of inferior PVR development [10–12]. In such eyes, the use of HSO has been suggested to be effective and safe for the treatment of inferior retina [8–14].

Most studies about the use of HSO in complex retinal detachments report that the HSO remained in the eye for an average of 3 to 6 months [2, 9, 10, 21, 22]. We studied eyes with a poor prognosis due to complex retinal detachments and extensive inferior PVR. Because of the severity of the retinal detachments, we left the HSO in the eyes for longer than 3 to 6 months and observed the effects of HSO over time.

Among the 25 eyes studied, the silicone oil has not been removed from 14 eyes due to the complexity of the cases. These patients had the HSO in situ for a mean period of 11.07 ± 7.44 months. The HSO was removed from 11 eyes after a mean of 26.55 ± 21.38 months. To our knowledge, such study is unique because no published studies have reported the effects of HSO in situ for as many months as in the current study.

Another factor in the current study that has not been reported in other series is the use of HSO in cases of tractional retinal detachments due to proliferative diabetic retinopathy with associated retinal tears and development of inferior PVR. We included eight patients with combined tractional and rhegmatogenous retinal detachment secondary to diabetic retinopathy. These eyes did not have higher complication rates compared with patients with a rhegmatogenous retinal detachment.

We observed a high anatomic success rate (defined in this current series as success until 6 months of follow-up) in eyes with primary complex retinal detachment and recurrent retinal detachment. Eight eyes in which HSO was used as a primary endotamponade agent had an anatomic success rate of 100%. Seventeen (68%) eyes had undergone a previous unsuccessful surgery for retinal reattachment and underwent a second surgery with injection of HSO. One eye had a persistent retinal detachment resulting from severe tractional retinal detachment secondary to diabetes retinopathy and PVR. Another eye had a recurrent rhegmatogenous retinal detachment despite retreatment and HSO tamponade. Thus, we achieved an anatomic success rate of 92% when we analyzed the data from the 25 eyes from this series.

Despite the final low VA due to the severity of the cases, there was statistical improvement in BCVA in the group that did not remove the HSO and in the group in which HSO was removed. The BCVA in the group in which HSO was not removed improved significantly during the first 6 months and remained stable until the end of follow-up. Among the eyes that had the oil removed, there was improvement in BCVA after 1 month which remained stable until the last visit (*P* < 0.05).

Previous studies have reported an intraoperative common complication related to an interaction between the HSO and perfluorocarbon. When these substances come into contact intraoperatively, a hyperviscous solution that is described as “sticky oil” forms [23]. Thus, direct perfluorocarbon-silicone exchange should not be performed. In the current study, three patients had a giant tear. In these cases, such as in all eyes submitted to intravitreal HSO injection, fluid-air exchange followed by injection of HSO was performed successfully.

Some authors consider HSO to be poorly tolerated intraocularly, leading to early removal of oil (3–6 months). The well-known effects of this tamponade agent are cataract formation, oil emulsification, ocular hypertension, proinflammatory response, macular ERMs, and high levels of intraocular pressure (IOP) [16]. A previous study that evaluated the tolerance and efficacy of Oxane HD as an internal tamponade for retinal detachment surgery reported that Oxane HD was well tolerated and did not appear to have proinflammatory effects [3].

A recent study analyzed 61 eyes and compared Densiron (Densiron-68, Fluoron Company, Neu-Ulm, Germany) and a normal density 1,000-centistoke silicone oil. The study reported similar complication rates of cataract formation, elevated IOP, inflammatory reaction, macular ERMs, and silicone oil emulsification [24].

We observed inflammation in the anterior chamber in 40% of eyes, which is similar to other reported studies in which Oxane HD was used [25]. Emulsification occurs earlier with HSOs (Oxane HD and Densiron) than with standard silicone oils [3, 8, 16]. In the current study, HSO emulsification occurred in 13 (52%) patients at a long-term follow-up. However, despite the HSO emulsification, these eyes required silicon oil tamponade to avoid PVR progression, hypotony, and globe atrophy. New interventions and silicon oil change may be also alternatives for management of such complications [3, 8]; however, the surgeon must be aware of the possibility of globe atrophy and BCVA decrease due to ischemic optic neuropathy [16]; so the risks versus benefits may be analysed before such decision for each specific case.

Similar to other studies, the IOP was elevated in 16% of patients in whom Oxane HD was injected [2, 11, 13]. Despite previous reports of high rates of IOP elevations in patients injected with HSO, the most recent data showed equivalent rates of IOP elevation when Oxane HD and Densiron were compared with standard silicone oil [24, 25].

## 5. Conclusion

In summary, this retrospective study found that HSO is an effective tamponade agent in both complex rhegmatogenous and tractional retinal detachments complicated by PVR.

Most patients had a good anatomic success rate with improved vision. Despite the high rates of HSO emulsification, it is possible to maintain the HSO in eyes for long periods with relative tolerability and safety resulting in useful vision for specific cases.

## Figures and Tables

**Table 1:** Physical properties of Oxane HD.

	Viscosity 3,800 centistokes (3,300 mPas at 25°C)
	Density 1.03 g/cm^3^ at 25°C
	Refractive index 1.40
	Volatility <0.1%
	Surface tension >40 mN/m
	RMN3 volume 11.9

**Table 2:** Pre- and postoperative records per patient.

Patient	Indication	Age/ gender	Lens status	Follow-up (months)	TA in the 1st surgery	PVR	TA in retreatment	Baseline VA (Snellen)	Final VA (Snellen)	Baseline IOP (mmHg)	Final IOP (mmHg)	Biomicroscopy findings	Time of HSO removal (months)	Redetachment	Comments
1	RRD	61/M	Pseudo	22	1000 cts SO	CP6	Oxane HD	20/200	20/40	16	14	Inflammatory cells in the AC, 1st month, KP in endothelium,6th month	6	No	Oil emulsification
2	RRD	53/F	Pseudo	18	C3F8 gas	CP3	Oxane HD	HM	HM	15	15	Normal	36	No	Ischemic optic nerve neuropathy; oil exchange due to IOP elevated; oil emulsification
3	RRD	57/M	Phakic	24	C3F8 gas	CA3	Oxane HD	HM	20/200	12	12	Inflammatory cells in the AC, 1st month	11	No	Ischemic optic nerve neuropathy; oil emulsification
4	RRD	63/F	Phakic	30	1000 cts SO	CP2	Oxane HD	LP	HM	10	13	Oil in the AC, 6th month	36	No	Oil emulsification
5	RRD	28/M	Phakic	36	C3F8 gas	CA6	Oxane HD	20/400	20/200	16	15	KP in endothelium and cells in the AC, 1st month	17	No	IOL fixation
6	RRD	22/F	Phakic	42	Oxane HD	CP1	NA	20/200	20/25	15	9	Normal	48	No	RD secondary to Toxoplasmosis
7	RRD	80/F	Phakic	24	5000 cts SO	CP8	Oxane HD	HM	CF 2m	17	16	KP in endothelium and cells in the AC, 1st month	NR	Yes	Giant tear; redetachment; oil emulsification
8	RRD	66/M	Phakic	24	Oxane HD	CA3	Oxane HD	HM	20/63	10	12	Normal	8	No	Epiretinal membrane; oil emulsification
9	RRD	62/M	Phakic	70	1000 cts SO	NA	Oxane HD	20/400	20/100	14	12	Normal	72	No	Oil exchange due to IOP elevated; oil emulsification
10	RRD	41/M	Phakic	6	Oxane HD	CP3	NA	CF 3m	20/80	13	13	Normal	NR	No	
11	RRD	66/M	Pseudo	12	5000 cts SO	CA10	Oxane HD	20/200	20/80	21	12	Normal	NR	No	
12	RRD	21/M	Phakic	12	Oxane HD	CA3	NA	HM	20/200	15	16	Inflammatory cells in the AC, 1st month	NR	No	RD secondary to open ocular trauma
13	RRD	62/M	Pseudo	12	Oxane HD	CP3	Oxane HD	20/200	20/80	14	17	Inflammatory cells in the AC, 1st month	NR	No	Oil emulsification
14	RRD	68/M	Pseudo	6	1000 cts SO	CP2	Oxane HD	CF 1m	20/40	18	18	Inflammatory cells in the AC, 1st month	NR	No	
15	RRD	54/M	Phakic	6	1000 cts SO	CP1	Oxane HD	CF 3m	20/32	24	17	Inflammatory cells in the AC, 1st month	NR	No	
16	RRD	20/F	Phakic	8	1000 cts SO	CP3	Oxane HD	LP	CF 1m	22	14	Normal	NR	No	Von Hippel-Lindau
17	RRD	64/M	Phakic	7	1000 cts SO	CP8	Oxane HD	CF 1m	20/80	23	13	Inflammatory cells in the AC, 1st month	NR	No	Intraoperative bleeding in the first surgery
18	TRD	56/M	Phakic	20	C3F8 gas	CP4	Oxane HD	CF 1m	20/80	13	20	Normal	6	No	Oil emulsification
19	TRD	24/M	Pseudo	44	C3F8 gas	NA	Oxane HD	HM	CF 3m	14	14	Normal	46	No	Optical nerve atrophy; oil emulsification
20	TRD	49/M	Pseudo	34	Oxane HD	CA10	5000 cts SO	LP	HM	14	15	Normal	6	No	Oil exchange due to IOP elevated; oil emulsification
21	TRD	63/F	Phakic	13	1000 cts SO	CA10	Oxane HD	LP	HM	12	14	Normal	NR	Yes	Ischemic optic nerve neuropathy and persistent retinal detachment
22	TRD	29/M	Pseudo	36	Oxane HD	CA1	NA	HM	20/400	18	11	Normal	NR	No	Oil emulsification
23	TRD	17/M	Pseudo	12	1000 cts SO	CA3	Oxane HD	CF 1m	20/80	17	10	Oil emulsification, 6th month	NR	No	Oil emulsification
24	TRD	39/M	Phakic	12	Oxane HD	CA10	NA	LP	HM	11	20	Normal	NR	No	Ischemic optic nerve neuropathy
25	TRD	60/M	Phakic	6	1000 cts SO	CP3	Oxane HD	CF 1m	20/200	25	18	Inflammatory cells in the AC, 1st month	NR	No	

RRD = rhegmatogenous retinal detachment; TRD = tractional retinal detachment; F = female; M = male; TA = tamponade agent; PVR = proliferative vitreoretinopathy; VA = visual acuity; IOP = intraocular pressure; SO = silicone oil; HSO = heavy silicone oil; HM = hand motion; CF = count fingers; LP = light perception; AC = anterior chamber; KP = keratic precipitates; IOL = intraocular lens; RD = retinal detachment; NR = not removed, Pseudo = pseudophakic; NA = not available; cts = centistokes.

**Table 3:** Comparison between LogMAR BCVA at baseline, month 1, month 6, and final in patients in whom HSO was removed and patients in whom HSO was not removed.

	Baseline	Month 1	Month 6	Final
HSO not removed (*n* = 14)				
Mean	1.90	1.22	1.07	1.03
SD	0.58	0.58	0.63	0.66
Median	1.79	1.15	0.80	0.80
Min	1.00	0.60	0.49	0.20
Max	2.70	2.20	2.20	2.20

HSO removed (*n* = 11)				
Mean	1.87	1.33	1.24	1.11
SD	0.63	0.74	0.80	0.79
Median	2.20	1.00	1.00	1.00
Min	1.00	0.30	0.20	0.09
Max	2.70	2.20	2.20	2.20

Patients in whom HSO was not removed.

Baseline > month 1 (*P* = 0.001), month 6 (*P* = 0.001), and final (*P* = 0.001).

Month 1 > month 6 (*P* = 0.027), final (*P* = 0.027).

Month 6 = final (*P* = 0.180).

Patients in whom HSO was removed.

Baseline > month 1 (*P* = 0.008), month 6 (*P* = 0.008), and final (*P* = 0.005).

Month 1 = month 6 (*P* = 0.068); month 1> final (*P* = 0.028).

Month 6 = final (*P* = 0.068).
